# Psychological foundations of digital empowerment: development and preliminary validation of self-determination theory–based scale for female university students

**DOI:** 10.3389/fpsyg.2026.1939454

**Published:** 2026-09-22

**Authors:** Afnan Fahad Bin Dayel

**Affiliations:** Department of Psychology, College of Social Sciences, Imam Mohammad Ibn Saud Islamic University (IMSIU), Riyadh, Saudi Arabia

**Keywords:** digital empowerment, female university students, psychometric properties, scale development, self-determination theory

## Abstract

**Inroduction:**

As higher education undergoes rapid digital transformation, digital empowerment has emerged as a pivotal psychological resource for student functioning. Existing instruments conceptualize empowerment narrowly as technical proficiency, and no psychometrically validated Arabic-language measure exists. Addressing both gaps, this study developed and preliminarily validated a Digital Empowerment Scale (DES) grounded in Self-Determination Theory.

**Methods:**

A sample of 248 female university students in Riyadh, Saudi Arabia (M age = 21.0, SD = 2.85) completed a 19-item pool tapping psychological competence and autonomy, technical-cognitive competence, relatedness, and digital, ethical, and legal awareness. Given marked non-normality, confirmatory factor analyses employed WLSMV estimation.

**Results:**

A four-factor solution showed poor discriminant validity between the psychological and technical factors (HTMT = 0.92); a respecified three-factor model, after removal of one weak item, fit the data very well (CFI = 0.961, TLI = 0.954, RMSEA = 0.037, SRMR = 0.079). The final 18-item scale demonstrated adequate composite reliability (ω = 0.66–0.76) and clear discriminant validity (HTMT ≤ 0.43).

**Discussion:**

Pending criterion-related evidence and cross-validation in independent samples, the DES offers a parsimonious, theoretically anchored preliminary instrument for assessing digital empowerment among female university students.

## 1 Introduction

The integration of digital technologies into higher education has gradually shifted attention from whether students can access technology toward how they might use it purposefully, autonomously, and responsibly. This capacity, often termed digital empowerment, has come to occupy an increasingly central place in contemporary academic life, and it may be understood as “the ability of individuals to use digital technology skillfully and efficiently to achieve their educational, professional, and social goals, in line with the requirements of the modern digital society” (Al-Surur and Al-Mubairik, 2026, p. 268). Yet this definition, like most in the field, tends to describe what individuals do with technology while remaining relatively silent on the psychological states that make such agency possible: the confidence, volition, and sense of connection that distinguish genuine empowerment from mere technical fluency. It is this psychological substrate that the present study seeks to examine.

The issue is far from academic for university students. Routinely described as “digital natives” by virtue of having matured amid the dominance of computers and networked systems (Prensky, 2001), these students are frequently presumed to be technologically proficient simply because they were raised in technology-rich environments. However, this presumption is misleading. Using digital tools for recreation or personal interest differs fundamentally from deploying them for learning, which demands sustained cognitive and emotional investment and may therefore appear less immediately appealing than recreational use (Wang et al., 2021). Digital empowerment is consequently not an inheritance of one’s birth cohort but a psychological achievement, one that some students attain and others do not.

Precisely because it is a psychological achievement, digital empowerment cannot be adequately understood through technical lenses alone. The construct is multidimensional, and literature reviews have shown that scholars have diverged markedly in specifying its components, a divergence that can be traced to differing scientific orientations and theoretical points of departure. Educational, administrative, and technical studies have each approached the construct from their respective perspectives, while research examining digital empowerment from a specifically psychological perspective remains limited, despite the pivotal role this dimension plays (Arif et al., 2024; Gömleksiz and Pullu, 2017; Irwansyah and Hardiah, 2020; Mäkinen, 2006; Tamang and Reddy, 2025).

This imbalance persists even though digital empowerment has been linked to positive psychological indicators, including the capacity to improve quality of life (Khatun and Yeasmin, 2026) and the way individuals think about themselves, their abilities, their potential, and the degree of control they exercise over what they possess (Irwansyah and Hardiah, 2020).

Self-Determination Theory (SDT) offers a principled means of redressing this imbalance. One of the most prominent motivational theories of our time, SDT holds that humans possess inherent basic psychological needs, autonomy (the experience of volition and willingness), competence (the experience of effectiveness and mastery), and relatedness (the experience of warmth, bonding, and care), that serve as the nutriments of motivation, growth, flourishing, and well-being; when these needs are satisfied humans thrive, whereas their frustration is associated with maladjustment (Olafsen et al., 2021; Ryan and Deci, 2000, 2017; Vansteenkiste et al., 2020). Notably, the theory treats each need as experientially distinct, and treats competence as a single, unitary need, one that it does not partition according to the domain in which effectiveness is exercised (Vansteenkiste et al., 2020). SDT has moreover proven particularly apt for technology-mediated contexts, owing to its interpretive flexibility, its capacity to accommodate diverse contextual factors, and its treatment of motivation as a multidimensional phenomenon (Wang et al., 2021). Viewed through this lens, digital empowerment can be reconceived psychologically as a construct encompassing autonomy, and relatedness together with competence, which in the present study was initially operationalized through two facets, psychological competence and technical-cognitive competence, a distinction the present analyses would later put to the test (Ryan and Deci, 2017): autonomy affords volitional, individualized engagement with learning, whereas relatedness supports communication and collaborative work in digital environments. To these three needs a fourth, value-based component may be added “digital, ethical, and legal awareness,” since factors such as the ethical use of digital technologies and digital security are indispensable when assessing digital empowerment across different segments of society, fostering critical thinking and responsibility within the digital environment (Tamang and Reddy, 2025). Similar measurement gaps have been documented for other emerging digital constructs; even for AI literacy, psychometrically validated instruments remain limited amid persistent debates over conceptualization and measurement (Liu et al., 2026).

The absence of a shared theoretical framework also explains the variation among existing instruments, as well as their common neglect of the psychological dimension. Mäkinen (2006) operationally defined digital empowerment through five components: awareness, motivation, technical access, competence, and constructive participation. Similarly, Tamang and Reddy (2025) measured this construct through technological competence, technical access, digital collaboration and communication, digital security, and the ethical use of technological resources. Even the most recent conceptualizations of digital empowerment have described the construct in terms of the effective integration of digital technologies in education, digital communication, digital evaluation, digital institutional support, technological motivation, and social networking empowerment (Jiménez Cortés, 2025). Despite the value of these scales, none of them addresses the psychological dimension in sufficient depth; even when it is nominally included, its treatment remains limited. Therefore, there is a need for an instrument grounded in Self-Determination Theory (SDT) that captures the psychological effects associated with engagement with technology and contributes to assessing its impact on behavior and mental health.

Two further gaps sharpen the case for the present study. First, no Arabic-language study, to the author’s knowledge, has developed a digital empowerment scale grounded in Self-Determination Theory, making such an instrument an urgent necessity for understanding the psychological consequences of constructive technology use for the psychological adjustment and mental health of university students amid digital transformation. Second, the focus on female university students is itself warranted: universities are pivotal in preparing human capital for the demands of the digital era. Yet, as indicated by the findings of Hossain et al. (2023), statistically significant differences were found between male and female students in digital competence, with male students demonstrating higher levels of competence. This finding underscores the need for studies that diagnose areas of weakness and shortfalls, particularly among female students.

Accordingly, the present study develops and undertakes a preliminary psychometric validation of a Digital Empowerment Scale (DES) for female university students, grounded in SDT and extended with a value dimension. Following established practice in the psychometric validation of SDT-based instruments (De Francisco et al., 2018; Olafsen et al., 2021), the researcher examines the scale’s internal consistency, factorial structure, convergent validity, and discriminant validity, the latter assessed through average variance extracted and heterotrait–monotrait ratios, alongside multiple reliability coefficients, using an estimator appropriate for ordinal, non-normal responses. In doing so, this study makes three contributions. First, this study will provide a theoretically anchored, Arabic-language instrument that diagnoses the degree to which female university students possess psychological–technical competence, relatedness, and digital, ethical, and legal awareness, thereby supporting their psychological adjustment to the demands of the digital environment. Second, this study offers empirical evidence bearing directly on SDT’s structure: the analyses reveal that the hypothesized psychological and technical facets are empirically inseparable, converging on a single psychological–technical competence dimension alongside distinct social and value dimensions, a finding consistent with SDT’s premise that perceived competence is not domain-bounded. Third, this study supplies a concise, reproducible measure suitable for research and for the design of programs and interventions that strengthen female students’ capacity to adapt to contemporary educational demands.

Based on this, the primary objective of this study is to develop and preliminarily validate a digital empowerment scale for female university students grounded in Self-Determination Theory.

## 2 Materials and methods

### 2.1 Measure development and analysis

Building on the digital-empowerment literature and self-determination theory (Mäkinen, 2006; Ryan and Deci, 2017), items were developed deductively in English and iteratively refined through rewording to a set of 19 items representing the hypothesized dimensions of digital empowerment: psychological autonomy and psychological competence constitute the psychological dimension (PSY); technical-cognitive competence constitutes the technical dimension (TECH); relatedness constitutes the social dimension (SOC); and digital, ethical, and legal awareness constitute the value dimension (VAL). Each item was rated on a five-point Likert scale (1 = never, 2 = rarely, 3 = sometimes, 4 = often, 5 = always), so that total scores could range from 19 to 95, with higher scores reflecting greater digital empowerment.

The scale was then translated and culturally adapted into Arabic in accordance with international guidelines for test translation and cross-cultural adaptation, with particular attention to conceptual and semantic equivalence (American Educational Research Association, American Psychological Association, National Council on Measurement in Education, 2014; Beaton et al., 2000; International Test Commission, 2017). First, two independent forward translations were conducted by bilingual (Arabic-English) translators familiar with psychological and psychometric terminology. Second, the two Arabic versions were compared item by item during a reconciliation process, discrepancies were resolved, and a single unified wording was agreed upon based on clarity, accuracy of meaning, conceptual equivalence, and appropriateness for female university students.

Third, to establish content validity, the unified Arabic version was evaluated by five expert judges in psychology and psychometrics at Imam Mohammad Ibn Saud Islamic University, purposively selected for their experience in developing and evaluating psychometric instruments; all held doctoral degrees and had more than 15 years of academic and professional experience. Each expert independently rated the 19 items for relevance to the intended construct, appropriateness for the target population, linguistic clarity, and cultural appropriateness, and provided qualitative comments on item wording. Agreement was calculated for each item using simple percentages, consistent with Koller et al. (2017), and ranged from 80 to 100%, corresponding to agreement among four to five of the five experts, indicating a high degree of consensus regarding the adequacy and relevance of the items. Based on the experts’ evaluations, necessary revisions were made to improve clarity, appropriateness, and relevance while preserving the intended meaning of the items, and the panel size is consistent with methodological recommendations that content validity be established by a minimum of five expert reviewers (Haynes et al., 1995; Koller et al., 2017).

Fourth, a blind back-translation into English was conducted by an independent bilingual translator who had not had access to the original English version. The back-translated version was compared with the original to identify semantic deviations, and where differences were identified, the corresponding Arabic items were revised iteratively until conceptual and semantic equivalence was achieved.

Finally, a pilot study was conducted with 5 female university students from the target population to assess the clarity and comprehensibility of the instructions and items before formal administration. The pilot indicated that the items were clear and easily understood, with no reported difficulties concerning comprehension or the response process; accordingly, no further modifications were necessary, and the final Arabic version was retained for the main study.

For the main study, the following analytic strategy was specified. Item distributions would first be examined, with factorability assessed using polychoric correlations together with the Kaiser-Meyer-Olkin measure and Bartlett’s test of sphericity, and the hypothesized structure would then be tested through confirmatory factor analysis with a robust estimator appropriate for ordinal data (WLSMV). Convergent and discriminant validity would be evaluated via composite reliability (ω), average variance extracted (AVE), and the heterotrait-monotrait (HTMT) ratio, while reliability would be assessed using Cronbach’s alpha, McDonald’s omega, and split-half coefficients. The design of the main study, the data collection procedures, and the characteristics of the sample to which this strategy was applied are described next.

### 2.2 Design, data collection, and participants

The present study employed a descriptive, cross-sectional, instrumental design to develop the Digital Empowerment Scale and evaluate its psychometric properties. The target population comprised female students at a public university in Riyadh, Saudi Arabia, during the second semester of 2025/2026. After data screening, 248 valid responses were retained (age range 18–35 years; M = 21.0, SD = 2.85). Demographic characteristics are presented in Table 1.

**Table 1:** Demographic characteristics of the sample (*N* = 248).

Variable	Category	*n*	%
Age	18–21 years	170	68.5
	22 years and older	78	31.5
Academic level	Bachelor’s	172	69.4
	Postgraduate	76	30.6
Total		248	100.0

Ethical approval was obtained from the university of the author of this study. All participants were informed about the purpose of the study and assured that the collected data would be anonymized and used solely for research purposes, and informed consent was obtained from all participants. Convenience sampling was used in this study. Participants were recruited from a public university in Riyadh, Saudi Arabia, through email and social media platforms, specifically WhatsApp. Female university students were targeted because the scale was developed for this population and in light of documented gender-related differences in digital empowerment (Hossain et al., 2023). Data were collected during the second semester of the 2025–2026 academic year using a self-administered online questionnaire created and distributed via Google Forms, as well as paper-based questionnaires distributed to female university students at the same university. Participation was voluntary and anonymous, and no identifying information (e.g., names, phone numbers, email addresses, or student IDs) was collected. The survey required approximately 10–15 min to complete. During data screening, all responses were reviewed for completeness. Two paper-based questionnaires were excluded because they contained incomplete data. After applying this exclusion criterion, the final sample consisted of 248 female university students.

The sample was considered adequate on three grounds: the participant-to-item ratio was 13:1 (248/19), exceeding the 10:1 benchmark commonly applied in factor-analytic research (Costello and Osborne, 2005); a recent validation study successfully applied WLSMV-based confirmatory factor analysis with a sample of 275 participants (Enko and Kościelniak, 2026); and individual validation samples of comparable size are common in SDT measurement research, with two of the four samples in Olafsen et al. (2021) comprising 281 and 299 participants.

## 3 Results

### 3.1 Item analysis and distributional properties

Item responses showed pronounced ceiling effects, with most items negatively skewed and several leptokurtic (e.g., item 16: skewness = −5.40, kurtosis = 33.56), indicating clear departures from normality. Accordingly, ordinal-appropriate estimation was used throughout: polychoric correlations for factorability and the robust weighted least squares mean- and variance-adjusted (WLSMV) estimator for confirmatory modeling. The data were suitable for factor analysis (KMO = 0.77; Bartlett’s test of sphericity χ^2^(171) = 959.48, *p* < 0.001).

### 3.2 Confirmatory factor analysis and model refinement

Because the scale was developed deductively from SDT, the hypothesized structure was tested confirmatorily on the full sample (*N* = 248) using WLSMV. The initial four-factor model (psychological, technical, social, value) fit the data adequately (Table 2); however, two items assigned to the technical factor loaded weakly (item 9, λ = 0.30; item 10, λ = 0.44), and the psychological and technical factors were nearly collinear (φ = 0.86; HTMT = 0.92), indicating inadequate discriminant validity between them.

**Table 2:** Model fit indices and comparison (WLSMV, *N* = 248).

Model	χ^2^ (scaled)	df	CFI	TLI	RMSEA	SRMR
Four-factor (initial)	201.835	146	0.953	0.945	0.039	0.082
Three-factor (PSY + TECH merged)	206.479	149	0.951	0.944	0.040	0.082
Three-factor, 18 items (final; i9 removed)	177.792	132	0.961	0.954	0.037	0.079

Scaled fit indices are reported. The final model omits item 9.

A more parsimonious three-factor model, in which the psychological items (including the autonomy items) and technical items loaded on a single latent factor (psychological–technical competence, hereafter PSY-TECH), was therefore tested. A scaled chi-square difference test indicated that the four-factor model did not fit significantly better than the three-factor model (Δχ^2^ = 5.49, Δdf = 3, *p* = 0.139), supporting the more parsimonious solution. Item 9 (“I handle minor technical problems on my own”) was then removed because its loading fell below the 0.40 retention criterion. Because item 9, although modeled within the technical factor, was worded in terms of acting autonomously, its removal left only two explicitly autonomy-oriented items (items 4 and 5), a limitation addressed in the Discussion, whereas the technical-cognitive content of the dimension remained fully represented by the retained items; the resulting final three-factor, 18-item model showed excellent fit (Table 2): CFI = 0.961, TLI = 0.954, RMSEA = 0.037 (90% CI [0.021, 0.051]), SRMR = 0.079. All standardized loadings were statistically significant and ranged from 0.41 to 0.85 (Table 3). The final measurement model, with standardized loadings, is depicted in Figure 1.

**Table 3:** Standardized factor loadings for the final three-factor model (*N* = 248).

Factor/item	λ (std.)
Psychological–technical competence (PSY-TECH)	
i1	0.702
i2	0.610
i3	0.663
i4	0.633
i5	0.601
i6	0.714
i7	0.570
i8	0.469
i10	0.407
Social (relatedness)	
i11	0.546
i12	0.566
i13	0.696
i14	0.802
Value (digital, ethical, and legal awareness)	
i15	0.654
i16	0.707
i17	0.759
i18	0.662
i19	0.848

All loadings are significant at *p* < 0.001. Item 9 was removed during refinement.

**Figure 1:**
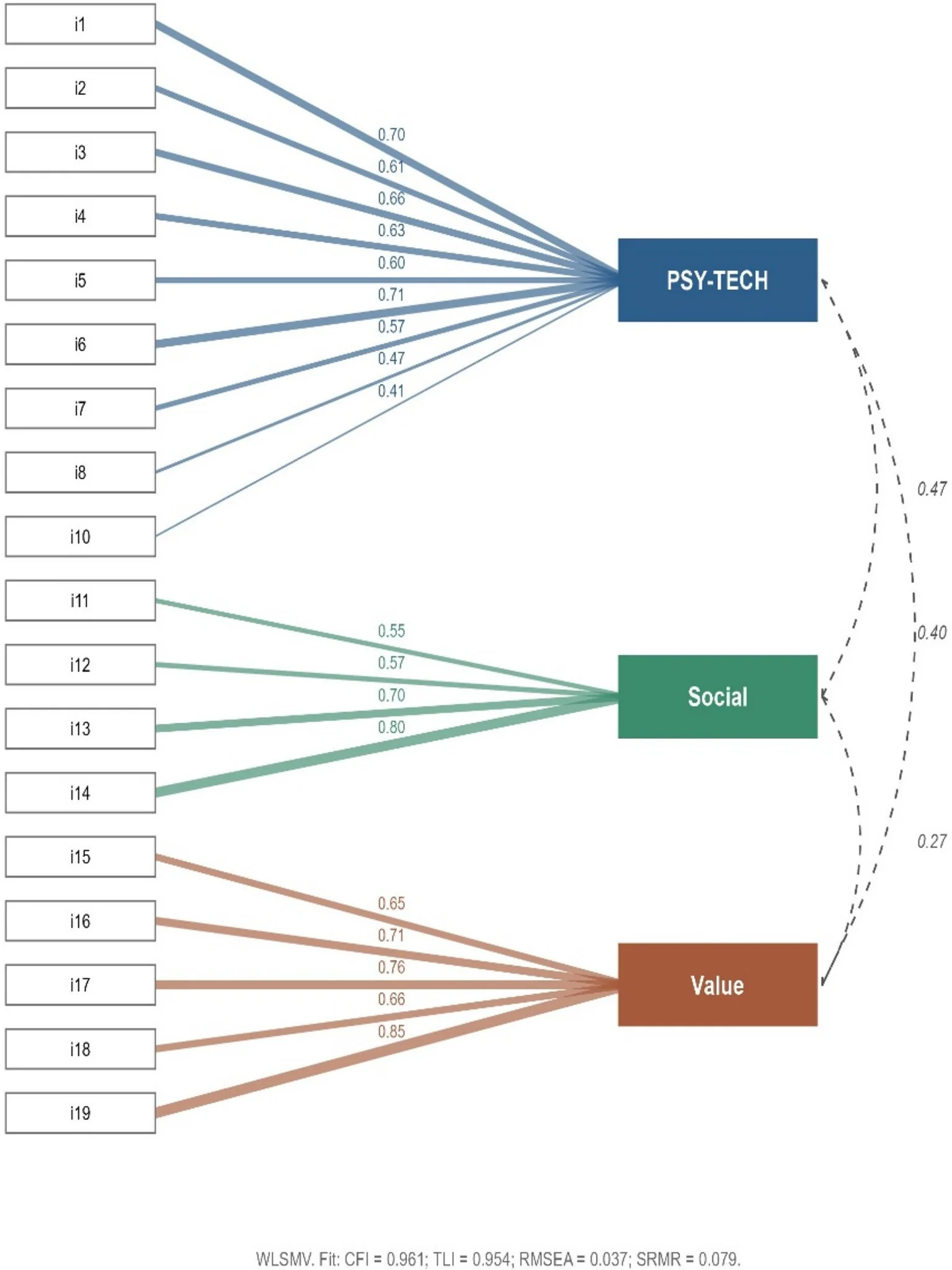
Confirmatory factor model of the final three-factor, 18-item Digital Empowerment Scale (standardized loadings; *N* = 248). PSY-TECH, psychological–technical competence.

### 3.3 Convergent and discriminant validity

Convergent validity was assessed through composite reliability (ω) and average variance extracted (AVE; Table 4). Composite reliability was 0.764 for the psychological–technical dimension and 0.727 for the value dimension, both exceeding the conventional 0.70 threshold, whereas the four-item social dimension fell slightly below it (ω = 0.664). AVE values were more modest, ranging from 0.365 (psychological–technical) to 0.532 (value). Only the value dimension met the 0.50 benchmark, whereas the psychological–technical and social dimensions (AVE = 0.365 and 0.437, respectively) fell below it. This pattern indicates that, although the items cohere reliably, they capture a relatively modest share of common variance in two dimensions, a limitation addressed in the Discussion. Notably, composite reliability remained close to or above the 0.70 threshold even where AVE was low, a divergence commonly observed with multidimensional scales and short subscales.

**Table 4:** Reliability and average variance extracted (*N* = 248).

Dimension	Items	α	ω (CR)	Split-half	AVE
Psychological–technical	9	0.750	0.764	0.800	0.365
Social	4	0.636	0.664	0.731	0.437
Value	5	0.711	0.727	0.754	0.532

Discriminant validity was clearly supported (Table 5). All heterotrait–monotrait (HTMT) ratios fell well below the conservative 0.85 criterion (Henseler et al., 2015), with the highest value reaching only 0.430 (psychological–technical with social). Consistent with this, the inter-factor correlations were low to moderate, ranging from 0.271 (social with value) to 0.466 (psychological–technical with social), confirming that the three dimensions, while related, are empirically distinct rather than redundant.

**Table 5:** HTMT ratios (below diagonal) and inter-factor correlations (above diagonal).

Dimension	PSY-TECH	Social	Value
PSY-TECH	—	0.466	0.403
Social	0.430	—	0.271
Value	0.386	0.259	—

HTMT < 0.85 indicates adequate discriminant validity (Henseler et al., 2015).

Internal-consistency reliability was likewise acceptable, as alpha values of 0.6–0.7 are generally regarded as indicating an acceptable level of reliability and values of 0.8 or greater a very good level (Ursachi et al., 2015; Winkler, 2022): Cronbach’s alpha was 0.750 (psychological–technical), 0.636 (social), and 0.711 (value), McDonald’s omega was 0.764, 0.664, and 0.727, respectively, and Guttman split-half coefficients were 0.800, 0.731, and 0.754 for the subscales. Reliability coefficients and HTMT ratios across the dimensions are summarized in Figure 2.

**Figure 2:**
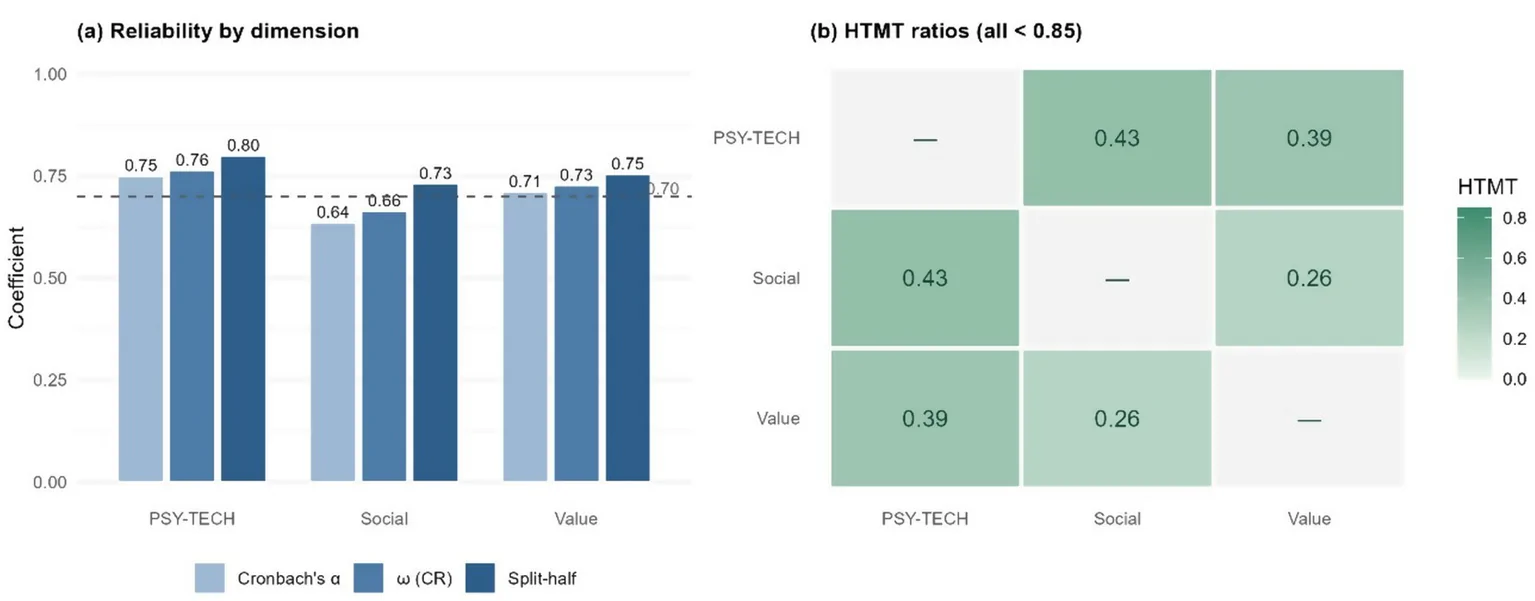
Reliability by dimension **(a)** and heterotrait–monotrait (HTMT) ratios **(b)** for the Digital Empowerment Scale (*N* = 248). All HTMT ratios are below the 0.85 criterion, supporting discriminant validity.

Given the moderate inter-factor correlations (0.27–0.47), dimension scores should be regarded as the primary units of interpretation; the total score may serve as a global summary index pending further evidence of a higher-order structure.

Taken together, the psychometric findings indicate that the 18-item Digital Empowerment Scale is reliable and structurally coherent. The three-factor model fit the data well, all items loaded significantly on their intended factors, internal-consistency and split-half coefficients were satisfactory, and the dimensions were empirically distinct. However, because average variance extracted fell below the 0.50 benchmark for two dimensions, convergent validity should be regarded as only partially established at this stage; the consistently adequate composite reliability and strong discriminant validity support the preliminary use of the scale as a concise, theoretically grounded measure of digital empowerment among female university students, pending cross-validation in independent samples.

## 4 Discussion

This study set out to develop and preliminarily validate a psychologically grounded measure of digital empowerment for female university students, and its central finding is as much conceptual as it is psychometric. The data did not support the hypothesized separation between a psychological dimension and a technical dimension. Rather than loading on distinct factors, items intended to capture psychological competence and autonomy on the one hand, and technical-cognitive competence on the other, proved empirically near-indistinguishable: the two latent factors correlated at 0.86 and yielded a heterotrait–monotrait ratio of 0.92, far exceeding conventional thresholds for discriminant validity (Henseler et al., 2015). A more parsimonious model merging them fit the data no worse than the four-factor specification, and a scaled chi-square difference test confirmed that the added complexity was not statistically justified.

The remaining structure was clear and robust. The value dimension, comprising digital, ethical, and legal awareness, emerged as the most strongly defined factor, reinforcing arguments that ethical and security considerations are integral to, rather than peripheral to, digital empowerment (Tamang and Reddy, 2025). The social dimension, capturing relatedness and constructive digital participation, was likewise well defined and consistent with accounts that foreground collaboration and belonging (Mäkinen, 2006), and with recent evidence linking basic psychological need satisfaction including relatedness to lower maladaptive internet use among students in higher education (Hu et al., 2026). Across all three dimensions, composite reliability was adequate or close to the conventional threshold and discriminant validity was clearly established, with all heterotrait–monotrait ratios at or below 0.43, indicating that the final 18-item scale measures related but distinct facets of a coherent psychological construct.

The convergence of the psychological and technical-cognitive facets is theoretically coherent rather than anomalous, and it speaks to an open question within Basic Psychological Need Theory. BPNT defines competence as a single basic need, namely the experience of effectiveness and mastery, satisfied as one capably engages in activities and encounters opportunities for using and extending one’s skills (Vansteenkiste et al., 2020). The theory does not partition this need according to the domain in which effectiveness is exercised. In hypothesizing a psychological competence factor separable from a technical-cognitive one, the present study effectively posited a distinction that SDT itself does not make, and the data indicate that this distinction does not survive empirical scrutiny. A student’s confidence in directing her own learning and her confidence in handling digital tools appear to draw on the same underlying sense of effectance (Ryan and Deci, 2017).

A further nuance of the merged factor warrants acknowledgment. The psychological facet as operationalized here included items tapping volitional self-direction (items 4 and 5) alongside emotional confidence, so the resulting psychological–technical dimension absorbs autonomy-related content as well as competence. This should not be read as evidence that autonomy and competence are empirically indistinguishable: validation studies of dedicated need-satisfaction measures consistently recover them as separable, moderately correlated factors (e.g., a mean latent correlation of 0.43 across four samples in Olafsen et al., 2021). Rather, with only two explicitly autonomy-oriented items (items 4 and 5) remaining after the removal of item 9, embedded within a broader competence-oriented pool, the present design did not afford a fair test of a distinct autonomy facet. The dimension is therefore best interpreted as capturing agentic psychological–technical functioning rather than competence narrowly construed, and future refinements of the DES should include a fuller set of autonomy items to test whether a dedicated autonomy facet can be recovered.

The decision to model autonomy and psychological–technical competence within a single dimension is nonetheless theoretically defensible. Within SDT, autonomy denotes the experience of volition and self-direction in initiating and regulating behavior, whereas competence denotes the perceived capacity to act effectively and attain mastery (Ryan and Deci, 2000, 2017); in technology-related action these needs are functionally complementary, since self-directed engagement with digital tools presupposes both the volition to initiate it and the perceived capability to enact it effectively. Consistent with this reasoning, Bergman et al. (2025) operationalized autonomy and competence as a single latent construct, autonomy–competence satisfaction, and justified the combination as consistent with SDT and prior research. The merged dimension therefore reflects the conceptual complementarity of the two needs rather than their theoretical identity and is best read as capturing self-directed and effective functioning in digital contexts.

This finding bears on a research direction that Vansteenkiste et al. (2020) explicitly identify as a priority for BPNT. Rather than extending the shortlist of basic needs, they propose, the theory might be refined by differentiating facets within a single need, with the proviso that such facets should share a common foundation while also containing unique aspects, and they note that such differentiations could in principle be made for autonomy and competence. The present study offers preliminary, single-sample evidence bearing on that proposition within the digital domain. The result is instructive: the common foundation dominated, and the putatively unique aspects proved insufficient to sustain a distinction. Rather than fragmenting into domain-specific facets, competence in the digital context behaved as BPNT would predict of a unitary basic need. This outcome is consistent with the theory’s distinctness criterion, according to which a basic need must be associated with a qualitatively different set of experiences rather than being derivative of, or largely coextensive with, another (Vansteenkiste et al., 2020), a criterion that the psychological–technical split conspicuously failed to meet although replication in independent and larger samples is required before firm conclusions can be drawn.

There are two further implications. First, this finding suggests that the prevalent assumption of the separability of the psychological and technical aspects of digital empowerment in self-reports, embedded in many existing instruments, warrants re-examination. The merging of those aspects into one dimension is no loss to the concept but may represent more faithfully how digital empowerment is experienced. Second, the value aspect emerged as a separate dimension rather than as a facet of competence and autonomy or relatedness. This finding supports the assertion that awareness of digital, ethical, and legal aspects deserves explicit inclusion alongside the SDT needs (Tamang and Reddy, 2025) rather than being absorbed into them. That the final structure is three-factor is therefore not an artifact of psychometric trimming; it reflects the theory’s own architecture, with competence and autonomy combined, relatedness recovered as a distinct need, and a value component standing apart from both.

These theoretical considerations point to potential practical value, contingent on the further validation outlined below. The 18-item DES offers a concise, theoretically grounded tool that could assist counselors and educators in identifying students who possess technical access yet report lower psychological–technical competence, autonomy, or sense of connection needed to use technology effectively for learning, students who might otherwise be overlooked on the assumption that the digital generation is uniformly proficient. Its three-dimensional structure could further make it suitable as a pre–post measure for evaluating interventions, such as digitally delivered well-being programs for university students currently under development (Ruini et al., 2024), allowing resources to be directed toward the dimension in which students are weakest. Finally, the explicit value dimension may help institutions monitor and promote responsible digital conduct, including privacy protection, ethical behavior, and respect for intellectual property, a concern rarely captured by competence-only instruments among female university students. Arabic-speaking populations also remain markedly underrepresented in psychometric research, and a theoretically grounded instrument developed for this context widens rather than narrows the evidence base of the field. Until criterion-related evidence and cross-validation are available, however, such applications should be regarded as prospective rather than established.

### 4.1 Limitations and future directions

Several limitations qualify these conclusions. First, average variance extracted fell below the 0.50 benchmark for two dimensions, indicating modest shared variance with the latent factor even where composite reliability is adequate; item refinement could strengthen convergent validity. Second, responses showed pronounced ceiling effects, consistent with generally high self-reported empowerment but potentially attenuating variance. Third, because no external measures were administered, criterion-related validity could not be examined; linking DES scores to theoretically relevant outcomes such as psychological well-being, academic engagement, and technology anxiety, ideally within a nomological network tested through structural equation modeling, is the most important next step and the principal reason this validation is characterized as preliminary. Fourth, because model respecification was conducted on the same sample used to evaluate the final model, the fit indices may capitalize on chance, making cross-validation in an independent sample essential. Finally, the sample comprised female students at a single Saudi university recruited non-probabilistically; the structure should therefore be cross-validated in independent, more diverse samples, its measurement invariance examined, and, as with comparable context-situated validations in this field, its cross-cultural validity across other Arabic-speaking and non-Arabic populations established. Because the DES operationalizes basic psychological needs that SDT posits as universal, it lends itself naturally to broader application; future research could examine its measurement invariance and validity across varied settings, including male, non-Saudi, and non-Arabic-speaking populations. Each of these limitations, however, marks a tractable next step rather than a barrier. With a preliminary structure now established, the DES can now be linked to the psychological outcomes it was designed to illuminate.

## 5 Conclusion

The DES is a parsimonious 18-item preliminary instrument for assessing digital empowerment among female university students within an SDT framework, with evidence of a coherent three-factor structure, adequate reliability, and clear discriminant validity. Its principal contribution is conceptual as much as psychometric: the empirical convergence of psychological and technical competence, alongside distinct social and value dimensions, suggests that digital empowerment is experienced as agentic engagement with technology rather than as technical skill alone. With cross-validation in independent samples and the addition of criterion-related evidence, the scale is positioned to support research on the psychological consequences of technology use and, prospectively, the identification of students who possess technical access yet report lower psychological–technical competence, autonomy, or sense of connection, the evaluation of interventions through pre–post assessment across its three dimensions, and the monitoring of responsible digital conduct.

Although developed in Arabic for a Saudi context, the DES operationalizes basic psychological needs that SDT posits as universal, and, like other SDT-based measures that have been adapted across languages, it can be translated and tested in other cultural settings. Because the DES is brief and available in Arabic, it is also well suited to research and institutional use in Arabic-speaking higher-education contexts, supporting national digital-transformation goals and enabling cross-study comparison once cross-validated in broader and more diverse samples. With its item set and psychometric properties fully documented, the DES is offered as an open foundation for researchers seeking to link digital empowerment to well-being and educational outcomes in Arabic-speaking contexts and beyond.

## Data Availability

The Digital Empowerment Scale (DES), in both its English and Arabic versions, is openly available in the Supplementary material of this article. The raw dataset supporting the conclusions of this article will be made available by the author upon reasonable request.
