# Supplementary material for: Psychological foundations of digital empowerment: development and preliminary validation of self-determination theory–based scale for female university students
Source: Front Psychol. 2026 Sep 22;17:1939454. doi: 10.3389/fpsyg.2026.1939454 (PMC13638590; doi:10.3389/fpsyg.2026.1939454)
Supplement: Supplementary file 1 [file Supplementary_file_1.pdf]

Supplementary Material

Digital Empowerment Scale (DES) — Final Items

Response scale: 1 = never, 2 = rarely, 3 = sometimes, 4 = often, 5 = always.

Psychological–technical competence (PSY-TECH; items 1–8, 10)

1. I trust my ability to use digital technology in learning without fear or hesitation.
2. I can learn new digital skills without becoming frustrated during the process.
3. Anxiety does not affect my performance when using technology.
4. When I face difficulty using technology, I make my own decisions to complete the task.
5. I decide for myself how to accomplish my tasks using technology, without direction or imposition from others.
6. I understand how to make use of educational applications and platforms in my learning.
7. I understand how to search for digital information effectively.
8. I can judge the quality of the electronic sources used in my coursework.
10. I can understand new technical instructions and apply them successfully.

Social dimension (relatedness and digital participation; items 11–14)

11. Discussions on educational platforms give me the opportunity to communicate with my classmates and exchange views.
12. Technology helps me maintain continuous communication with my classmates while carrying out collaborative projects.
13. Expressing my ideas and opinions in digital environments helps me build positive interaction with others.
14. Interacting with others through digital environments gives me a sense of connection and belonging.

Value dimension (digital, ethical, and legal awareness; items 15–19)

15. I recognize the importance of privacy settings when using digital applications.
16. I recognize the importance of ethical conduct when using digital technologies (e.g., not harming others through words or images, and avoiding bullying).
17. I am aware that abuse, threats, or spreading misleading or harmful information online entails legal responsibility.
18. I am keen to respect intellectual-property rights when using digital content by citing sources.
19. I deal cautiously with websites and applications that request my data.

Note: Item numbers correspond to the original 19-item pool; item 9 was removed during model refinement (see Section 3.2).

مقياس التمكين الرقمي

ملاحظة: أرقام العبارات تطابق الصورة الأولية ذات ١٩ عبارة؛ حُذفت العبارة (٩) أثناء تنقيح النموذج (انظر القسم ٣,٢).

عزيزتي الطالبة، فيما يلي مجموعة من العبارات حول استخدامك للتكنولوجيا الرقمية. اقرئي كل عبارة وضعي علامة (✓) أمام الاستجابة التي تنطبق عليكِ. لا توجد إجابة صحيحة وأخرى خاطئة، والبيانات تُستخدم لأغراض البحث العلمي فقط.

العبارة	دائمًا	غالبًا	أحيانًا	نادرًا	أبدًا
**أولًا: البُعد النفسي: التقني (PSY-TECH)**					
١- أثق بقدرتي على استخدام التكنولوجيا الرقمية في التعلم دون خوف أو تردد.					
٢- أستطيع تعلم مهارات رقمية جديدة دون أن أحبَط أثناء التعلم.					
٣- لا يؤثر القلق في أدائي عند استخدام التقنية.					
٤- عندما أواجه صعوبة في استخدام التقنية، أتخذ قراراتي بنفسي لإكمال المهمة.					
٥- أحدد بنفسي الطريقة التي أنجز بها مهامي باستخدام التقنية دون توجيه أو فرض من الآخرين.					
٦- أفهم كيفية الاستفادة من التطبيقات والمنصات التعليمية في تعلمي.					
٧- أفهم كيفية البحث عن المعلومات الرقمية بشكل فعّال.					
٨- أستطيع الحكم على جودة المصادر الإلكترونية المستخدمة في واجباتي الدراسية.					

العبارة	دائمًا	غالبًا	أحيانًا	نادرًا	أبدًا
١٠- أستطيع فهم الإرشادات التقنية الجديدة وتطبيقها بنجاح.					
**ثانيًا: البُعد الاجتماعي (الارتباط بالآخرين والمشاركة الرقمية)**					
١١- تتيح لي النقاشات عبر المنصات التعليمية فرصة التواصل مع زميلاتي وتبادل الآراء معهن.					
١٢- تساعدني التكنولوجيا على التواصل المستمر مع زميلاتي في الدراسة أثناء تنفيذ المشاريع التشاركية.					
١٣- يساعدني التعبير عن أفكاري وآرائي في البيئات الرقمية على بناء تفاعل إيجابي مع الآخرين.					
١٤- يوفر لي التفاعل مع الآخرين عبر البيئات الرقمية شعورًا بالتواصل والانتماء.					
**ثالثًا: البُعد القيمي (الوعي الرقمي والأخلاقي والقانوني)**					
١٥- أدرك أهمية إعدادات الخصوصية عند استخدام التطبيقات الرقمية.					
١٦- أدرك أهمية الالتزام بالسلوك الأخلاقي عند استخدام التقنيات الرقمية. مثل: عدم إساءة الآخرين بالكلمات أو الصور والتنمر.					
١٧- أعي أن الإساءة أو التهديد أو نشر معلومات مضللة أو ضارة عبر الإنترنت يترتب عليه مسؤولية قانونية.					
١٨- أحرص على الالتزام بحقوق الملكية الفكرية عند استخدام المحتوى الرقمي من خلال توثيق المصادر.					
١٩- أتعامل بحذر مع المواقع والتطبيقات التي تطلب بياناتي.					

التصحيح: تدريج خماسي (دائمًا = ٥، غالبًا = ٤، أحيانًا = ٣، نادرًا = ٢، أبدًا = ١). تتراوح الدرجة الكلية بين ١٨ و٩٠، وتدل الدرجة المرتفعة على مستوى أعلى من التمكين الرقمي
